# Pathways and approaches for scaling-up of community-based management of acute malnutrition programs through the lens of complex adaptive systems in South Sudan

**DOI:** 10.1186/s13690-022-00934-y

**Published:** 2022-09-05

**Authors:** Andre M. N. Renzaho, Gilbert Dachi, Eric Ategbo, Stanley Chitekwe, Daniel Doh

**Affiliations:** 1 Translational Health Research Institute, School of Medicine, Western Sydney, University, Sydney, Australia; 2 Maternal, Child and Adolescent Health Program, Burnet Institute, Melbourne, VIC 3004 Australia; 3 UNICEF South Sudan; Totto Chan Compound, PO Box 45, Juba, South Sudan; 4 Nutrition Section, UNICEF Ethiopia, Addis Ababa, Ethiopia; 5 School of Social Sciences, Western Sydney University, Locked Bag 1797, Penrith, NSW 2751 Australia

**Keywords:** CMAM, Scaling-up, Community-based management of acute malnutrition, South Sudan

## Abstract

**Background:**

Funds for community-based management of acute malnutrition (CMAM) programs are short-term in nature. CMAM programs are implemented in countries with weak policies and health systems and are primarily funded by donors. Beyond operational expansion, their institutionalisation and alignment with governments’ priorities are poorly documented. The study aimed to identify pathway opportunities and approaches for horizontal and vertical scaling up of CMAM programs in South Sudan.

**Methods:**

The study was conducted in South Sudan between August and September 2021 using an online qualitative survey with 31 respondents from policy and implementing organisations. The Reach, Effectiveness, Adoption, Implementation, and Maintenance (RE-AIM) framework guided the study’s design. It was self-administered through the Qualtrics platform. We used Qualitative Content Analysis supported by the Nvivo coding process. A deductive a priori template of codes approach was complemented by a data-driven inductive approach to develop the second level of interpretive understanding.

**Results:**

Findings from the study demonstrate that the emphasis of CMAM programs was horizontal scaling up, characterised by geographic distribution and coverage as well as operational expansion. Main challenges have included unsustainable funding models, the inadequacy of existing infrastructure, high operational costs, cultural beliefs, and access-related barriers. Factor impacting access to CMAM programs have been geographical terrains, safety, and security concerns. Vertical scaling up, which emphasises institutional and ownership strengthening through a sound policy, regulatory, and fiscal environment, received relatively little attention. Nutrition supplies are not part of the government’s essential drug list and there is limited or no budgetary allocation for nutrition programs by the government in national budgets and fiscal strategies. Factors constraining vertical scalability have included weak government systems and capacity, a lack of advocacy and lobbying opportunities, and an apparent lack of exits strategies.

**Conclusion:**

Addressing the scalability problems of CMAM programs in South Sudan demands a delicate balancing act that prioritises both horizontal and vertical scalability. Government and political leadership that harness multidisciplinary and multi-sectoral coordination are required. There is a need to increase policy commitment to malnutrition and associated budgetary allocation, emphasise local resource mobilisation, and ensure financial sustainability of integrating CMAM programs into the existing health and welfare system.

**Supplementary Information:**

The online version contains supplementary material available at 10.1186/s13690-022-00934-y.

## Study highlight

**Table:** 

• CMAM programs are of utmost significance for children’s survival in emergencies. They are supported by well-developed sets of principles, protocols, and minimum humanitarian standards, • However, CMAM programs are implemented in countries with weak policies and health systems. They are primarily funded by external donors and their funding is short-term in nature. Their institutionalisation and alignment with governments’ priorities are poorly documented. • To maximise CMAM programs’ scalability in South Sudan, it is a policy imperative to ✓ advocate for strengthening domestic capacity and resource mobilization while leveraging external collaborations and support through a sliding scale of match funding percentages with domestic resources increasing with time as an exit strategy. ✓ institute a delicate balancing act that prioritises both horizontal and vertical scalability that considers context-specific factors and incremental scalability pathways. ✓ cultivate government and political leadership capable of harnessing multidisciplinary and multi-sectoral coordination ✓ Increase policy commitment to malnutrition and associated budgetary allocation, emphasise local resource mobilisation, and ensure financial sustainability by integrating CMAM programs into the existing health and welfare system.	

## Background

Whilst the term malnutrition encompasses both undernutrition and overnutrition (overweight and obesity), over the last 70 years the literature has predominantly used the term ‘malnutrition’ to refer to undernutrition. Therefore, in this paper we follow the same trend and use the term ‘malnutrition’ in lieu of ‘undernutrition’ [1]. Various definitions have been provided over the years, but strictly speaking malnutrition encompasses wasting (weight-for-height < − 2 Z-scores or bilateral oedema), stunting (height-for-age < − 2 Z-scores), underweight (weight-for-age < − 2 Z-scores) and inadequate intake of vitamins or minerals. To provide a more precise definition, Golden proposed the type I and type II nutrient deficiency typology [2]. A child with Type I deficiency continues to grow and uses up body stores of the nutrient itself to overcome any shortage. As a consequence, a reduction in bodily functions are accompanied with a decline in the metabolic function that depends on that nutrient. The child becomes ill, with clear clinical symptoms. Examples include micronutrient deficiencies such as iron deficiency (anemia), iodine deficiency (goitre), vitamin B 1 or thiamine deficiency (beri-beri), niacin deficiency (pellagra), vitamin C deficiency (scurvy), or vitamin A deficiency (xerophthalmia). In contrast, a child with Type II nutrient deficiency experiences growth failure and stops growing accompanied by weight loss. The body stops repairing tissue to conserve the nutrient in the body. The body breaks down its own tissues to make the nutrient available to the body. However, these nutrients control each other’s balance, meaning that a loss of one nutrient leads to a negative balance of the other type II nutrients, making it hard to diagnose any deficiency. Hence, there are no specific clinical symptoms, and only a dietary supplementation with a balance of nutrients will lead to rapid recovery. Examples of type II deficiency include potassium, sodium, magnesium, zinc, phosphorus, and proteins, which manifest into stunting, underweight, and wasting [2].

Globally, malnutrition remains a significant public health issue and unequally distributed, with its burden predominantly high in low and middle income countries (LMICs) where 99% of affected children live [3]. Overall, data from the Global Burden of Diseases indicated that the prevalence of child malnutrition in LMICs decreased between 2000 and 2017, declining from 36.9 to 26.6% for stunting, 8.4 to 6.4% for wasting, and 19.8 to 13.0% for underweight [3]. Although its preventable, malnutrition is associated with high morbidity and mortality [3]. Despite the observed progress, many LMICs will not meet the Global Nutrition Targets of reducing stunting by 40% and wasting to less than 5% by 2025 [3]. Approaches to treat malnutrition date back to the 1940s [4]. Early approaches in the 1940s and 1950s, considered malnutrition as a deficiency in energy and protein and emphasised that there was little evidence of the relationship between malnutrition and infection [5]. Hence, malnutrition was conceptualised as treatable by dietary supplement measures alone [5]. It was not until the 1960s that strong evidence on the malnutrition-infection vicious cycle emerged [4–8]. These findings shed light on the need for a complex management of malnutrition that incorporated dietary supplements and the treatment of infections. By the 1980s and 1990s, the synergism of nutrition, infection and immunity; and the role of micronutrient deficiencies in the pathogenesis of malnutrition became widely recognized [4, 5, 9]. These discoveries led to the formulation of comprehensive centre-based feeding approaches that combined dietary feeding, treatment of infections, and physical and psychological stimulation [10, 11].

Comprehensive centre-based feeding approaches evolved over the years, and were articulated by the 1981World Health Organisation (WHO)‘s manual on the ‘treatment and management of severe protein-energy malnutrition’ [10], and became widely used in emergency response to prevent avoidable malnutrition-related deaths [11]. In 1995, Médecins Sans Frontières (MSF) published the first nutritional guidelines outlining the planning, implementation and evaluation of centre-based management of child malnutrition in emergency settings [11]. These guidelines became instrumental in managing child malnutrition in emergency settings. Building on this success, the WHO published its protocols for managing severe child malnutrition in 1999 [12]. Children with malnutrition were admitted to therapeutic feeding programs, paediatric wards or even nutrition rehabilitation units for weeks and treated according to MSF nutritional guidelines and WHO protocols [11–13]. These centres were labour intensive and effective in managing child malnutrition. However, admitted children were accompanied by one or both parents away from the rest of the family [14]. Such an approach led to a number of serious challenges including low coverage rates, late presentation and associated complications, overcrowding and the risk of cross-infection, limited capacity as well as paucity of skilled staff and associated heavy staff workloads, high default rates, and possibly high-risk behaviours among mothers to cover meals [14–18].

Community-based management of acute malnutrition (CMAM) programs emerged as the best way to overcome these challenges. CMAM programs were piloted in 2000 and their evaluations found that they achieve better outcomes than traditional centre-based feeding programs [16, 19, 20]. They were subsequently endorsed by the United Nations in 2007 and gained widespread acceptance in humanitarian and non-humanitarian contexts across LMICs [21]. CMAM programs incorporate three components. The in-patient or stabilisation centre treats severely and moderately malnourished children (i.e. weight-for-height < − 2 Z-scores or bilateral oedema) with medical complications such as severe anaemia, lack of appetite, hypoglycaemia, diarrhoea with dehydration, hypothermia, and sever infections. Treatments include systematic medical treatment and milk-based formula such as F-75 (low protein and low energy milk at the beginning for the treatment) and F-100 (high protein and high energy when children regain appetite). The outpatient therapeutic programs (OTP) are for severely malnourished children (i.e. weight-for-height < − 3 Z-scores or bilateral oedema) without medical complications. They receive ready-to-use therapeutic foods (RUTFs), which are lipid-based therapeutic foods. The supplementary feeding programs are for moderately malnourished children (i.e. weight-for-height > − 3 but < − 2 Z-scores with no oedema) without medical complication. They get ready-to-use supplementary foods such as fortified blended foods or lipid-based nutrient supplements, which are dry take home supplementary rations [22]. The availability of ready to use therapeutic foods (RUTF) has made it possible to treat children in their homes [14, 19].

CMAM programs have been extensively evaluated to establish their effectiveness in Asia [23], Africa [14, 24, 25], and South America [20]. These evaluations have found CMAM programs to be effective in achieving good recovery and survival rates, and low defaulting and relapse rates. There is a strong and harmonised multiagency and multidisciplinary coordination of CMAM program implementation among United Nation agencies and international non-government organisations, and to some extent health ministries. Despite these successes, CMAM programs still draw insufficient attention for global implementation, hampering their integration into local or regional routine health systems [14, 24, 26]. They are often not integrated in national healthcare systems due to countries’ weak health systems, fragile health budgets that rely on external assistance, limited opportunities for competency-based learning and knowledge transfer, and the neglect in practice of the management of acute malnutrition in children below 6 months of age even when treatment protocols exist [14, 24, 26–28].

The scalability of CMAM programs remains poorly documented. South Sudan provides a timely case study to explore scalability pathways for CMAM program for many reasons. First, it is the youngest nation in Africa, with worrying levels of child malnutrition. Second, it receives generous funding from external donors to support various nutrition programs and coordination efforts. Thirdly, it joined the global the Scaling Up Nutrition (SUN) Movement in 2013 and expressed the political commitment to scale up nutrition interventions. Finally, by 2015, it had established the South Sudan SUN Movement Steering committee to verse the scalability efforts [29]. However, the term scale-up has been used in a variety of ways, but is often used to refer to “*deliberate efforts to increase the impact of successfully tested health innovations so as to benefit more people and to foster policy and program development on a lasting basis” (p.2)* [30]. It is beyond “doing more of” approach and encompasses horizontal scaling up based on intervention structures and three types of vertical scaling up based on intervention programs, strategies, and resource-base [30–34].

*Horizontal scaling* also known as quantitative scaling up or scaling out or simply scaling up by operational expansion is concerned with the growth or expansion of a successful intervention or policy [30–34]. It may involve the implementation of different distinctly or in combination. This may include the spread of the successful intervention or policy to cover more people and greater geographic space. It may emphasize replication of a successful intervention or policy elsewhere or nurturing of local initiatives through incentive-based approaches to integrate a successful intervention or policy. Or it may focus on aggregation of distinct organizations’ resources through horizontal processes that involve partial or full merger to streamline the expansion of a successful intervention or policy [35].

In contrast, v*ertical scaling up* or institutionalisation or mainstreaming focuses on the ownership of a successful intervention or policy in a variety of forms organisationally and functionally [30–34, 36]. For example, vertical integration may encompass integrating a successful intervention or policy into existing government structures and systems for a better coverage. It may involve increasing the scope of activities to add a new innovative intervention or policy to an existing package such as linking agriculture to CMAM programs to prevent and mitigate the early onset of malnutrition or relapse after a child had been discharged from the program (functional scaling up also known as diversification or grafting).. Vertical integration may equally focus on building a political power base to further the goals of a successful interventions or policy through political processes, reforms, and lobbying and advocacy (political scaling up or institutionalisation). This type of scaling up focuses on empowerment of people and institutions to facilitate needed structural and institutional changes to address contextual, social, political, economic, and environmental challenges that impede participation as well as the uptake of a successful intervention or policy. An example could be a civil society demanding that child malnutrition be seen as a child protection issue and embark of advocating that child protection measures be embedded into CMAM indicators. Similarly in some low and middle income countries, CMAM programs are not part of national health priorities. Political scaling up may involve advocating for the inclusion of CMAM programs into national nutritional protocols. Organisationally, vertical scaling up may emphasise improving organisations’ effectiveness, efficiency and sustainability through financial diversification (i.e. financial self-sufficiency), public-private partnership networking to improve internal management capacity (staff training and capacity building), and enacting public legislation to earmark entitlements within the annual budgets (organisational scaling up also known as institutional development) [33, 36].

There is international consensus that, whilst there is no hierarchy among these four types of scaling up, horizontal and vertical scaling need to go hand in hand for international aid programs, where program expansion and coverage must prioritise local ownership and capacities, incorporate program diversification to maximise synergy, and ensure institutionalisation through mainstreaming and context specific structural adjustments. In the case of CMAM programs, the emphasis has been on relying solely of external funding for horizontal scaling up, with most international non-government organisations (NGOs) significantly expand their geographic coverage, operational budgets, and staffing. However, pathways to CMAM programs’ integration into local and national social and health systems and the extent to which functional stovepiping threatens the long-term success of CMAM programs are poorly understood [34, 37]. Funds for CMAM programs are short-term in nature and implemented in countries with weak policies and health system, meaning that donors funds are also used to fund the alignment of donors’ priorities with beneficiary states’ policies and strategies [38]. This limits and threatens beneficiary governments’ critical direct involvement, making CMAM programs the responsibility of external donors. Undersetting factors that facilitate the alignment of CMAM programs with beneficiary governments’ priorities and associated capacity building and health system strengthening could be a key to achieving ownership and institutionalisation. Therefore, the aim of this study was to identify pathways opportunities and approaches for horizontal and vertical scaling up of CMAM programs in South Sudan.

## Methods

### Setting, study design and theoretical foundation

The study used an online qualitative survey, carried out in South Sudan between August and September 2021. South Sudan represents a complex adaptive system, which has been consistently dealing with challenges associated with successions of conflicts and political instability since its independence on 9 July 2011 [14, 39–41]. Outbreaks of new armed civil conflicts post-independence have escalated into conflicts between the various ethnic groups, leadership personalities centred on the struggle for political power and dominance, and armed factions [39, 42]. The consequences of such instability include weak state structures; stunted economic, social, and political progress; impaired capacity to build social protection blocks and political participation pathways that enable communities to improve and sustain their living conditions; and mass population displacements [39–41]. The simultaneous interactions and relationships of conflicts, community structures and the political landscape are affected and shaped by the system through CMAM programs are implemented.

The study design was informed by the Reach, Effectiveness, Adoption, Implementation, and Maintenance (RE-AIM) framework, guided the design of the online qualitative survey [43, 44]. The framework allowed the identification of barriers to and opportunities for CMAM programs’ expansion (i.e. geographic reach and the number of people served), effectiveness (i.e. the if, how, and why they improve recovery and survival and any heterogeneity in their impact), adoption (i.e. synergy at setting levels and of human resources), implementation (i.e. consistency, fidelity, and affordability), and maintenance (i.e. long-term impact and setting capacity for their institutionalisation). Although qualitative research has predominantly emphasised face-to-face interactions, the COVID-19 has highlighted the importance of online interactions in qualitative research. The use of email attachments or video chat features such as Skype and Zoom allow researcher to conduct online personal interviews and record such interviews for further analyses using traditional qualitative approaches. However, in countries like South Sudan, the internet is unreliable, often with poor internet coverage in rural areas and connectivity overall (slow speeds); and frequent disruptions by the government due to insecurity as well as to prevent activists from protesting against political machineries in the country. The time difference between Sydney, Australia, and South Sudan (− 9 hours) was another challenge to video chat features.

Therefore, the online qualitative survey was implemented using the Qualtrics platform and was self-administered. This approach has recently been advocated for and successfully implemented in various studies [45–47]. Advantages of this approach are multidimensional [45, 46]. There are no bleary-eyed video calls very early in the morning or very late in the night. There are no transcription costs. There are fewer ethical concerns as there is no direct interaction with participants. Participants can engage with the online survey when it is most convenient for them to do so and in several short bursts according to internet availability, time schedule, and commitments. Participants can reconstruct and express the contextual and political realities of CMAM program implementation freely on their own terms and comprehensively without narrowing their responses. The approach increase coverage as it increases access to participants who are geographically dispersed and/or experiencing physical or psychological difficulties that make participation in face-to-face data collection difficult (as most CMA programs in South Sudan are mainly in rural areas with poor internet coverage and connectivity).

### Recruitment and data collection

At the time of the online qualitative survey, there were 50 agencies involved in the implementation of CMAM programs across South Sudan. These included five United Nations agencies (e.g. World Food Program, the United Nations High Commissioner for Refugee, the United Nations Children’s Fund, and the Food and Agriculture Organization of the United Nations), 28 international NGOs, 16 local NGOs, and the South Sudan Ministry of Health involved. The online qualitative survey link, which contained the participant information sheet, online consent process, and the interview guide, was sent to all 50 agencies with an invitation to participate. A total of 31 organizations across South Sudan responded to and participated in the study. All participants provided online informed consent prior to participating in the study. The Qualtrics platform allowed respondents to provide detailed qualitative responses (textual data) to specific questions informed by the RE-AIM framework relating to the scalability of CMAM program including CMAM program impact, the involvement of local community-based structures and their capacity, funding mechanisms, and system integration and competencies. Other issues examined centred on the roles of key actors, factors facilitating or mitigating the use of RUTF across the country. CMAM programs in South Sudan are implemented along the funder and policy maker /implementing agency dichotomy. That is, United Nations agencies provide technical coordination, support the development of guidelines and policies, coordinate technical working group, oversee implementation activities and joint field visit, and raise funds for CMAM programs through funding advocacy initiatives. The ministry of health develop policies and guidelines and leads technic working groups and oversight. NGOs implement the oversee CMAM programs’ implementation and evaluation. Therefore, the interview guide was customized so that questions were specific to funding and policy level participants or CMAM program implementation and delivery level (Appendix 1).

### Data analysis

Qualitative Content Analysis was our preferred analytical approach [46, 48]. There is no doubt that research participants’ explanation of the implementation issues and scalability of CMAM programs was based on their interaction with the programs per se, programs beneficiaries and their families, the community at large and its structures, government institutions, and donors. The accounts were also based on the subjective explanation of their experiences and observations of program operations. To facilitate an extensive narrative of these interactions and observations, we used a directed content analysis of textual data incorporating two approaches: deductive (closed) and inductive (open) coding procedures [49]. The deductive coding developed a codebook using pre-determined key concepts to guide coding process. For the inductive coding procedure, textual data that could not fit into the predetermined categories were further analysed to identify new categories [5].

A deductive a priori template of codes approach was complemented by data-driven inductive approach to develop the second level of interpretive understanding [50]. Predetermined codes were generated in two ways: We carried out a study that examined the impact of CMAM programs in South Sudan and identified paths to CMAM program scalability [14]. Data from the study were complemented by RE-AIM framework [43, 44]. and the literature [30, 32, 34] to identify key concepts related to scaling up as coding categories. Operational definitions of these key concepts were:

- CMAM program impact through co-operation and cultivating government relations
- political and economic space (institutionalisation of CMAM programs, government budgets, and working with and within government structures to influence policy and systems)
- CMAM program diversification and synergy: costs and resource mobilisation
- cultural space and organizational process for CMAM program operational expansion (pace of expansion, decision-making processes, funding models, capacity and relationship building, local buy-in and cultural fit, number of organisations involved, whether centralised vs decentralised or adaptive vs fixed expansion)
- capacity and partnership space for lobbying and advocacy (alliances and partnerships, build constituencies within institutions with shared vision and ideologies, human resource development, and networking to strengthen community-level initiatives)
- learning space and knowledge mobilisation: evidence-informed scaling up, cultivating champions, and gatekeepers, and dissemination and drawing from lessons learned

We supported the coding and analysis process with the NVIVO software. Coding occurred in three stages. The first stage involved both the Principal Investigator (PI) and UNICEF South Sudan (second and third authors) defining predetermined key concepts collaboratively to facilitate the deductive coding process while also allowing for new ideas through inductive coding. External input was sought from an experienced nutritionist from UNICEF Ethiopia who has overseen the scaling up of CMAM programs in Africa as well as South Asia (fourth author). Following that, a research officer (last author) coded the full data set in the second stage, with the coding process stages and outcomes reviewed by the research team for consistency and accuracy. Finally, the research officer and the research team discussed the emerging themes and sub-themes, ensuring that they were consistent and aligned across the data. Overall, six processes were followed in the data analysis: a close reading of the textual data for familiarity; the generation of first codes; the search for themes; the review of initial themes; defining and naming the themes, and the preparation of the final findings. The results are presented in two ways: the quantification of the emerging codes through frequency and respective percentage in order to derive a rough indication of the potential importance of the factors influencing CMAM program scalability in South Sudan and the incorporation of open codes into refined ones as representative statements to allow a narrative layering and textual meaning. This approach is recommended in directed qualitative content analyses [51–54]. Respondents were deidentified using the codes FR1 to FR31 (where “FR” is Field Respondent) and their level of involvement either as implementers or as policy.

## Results

Respondents from 31 (16 men and 15 women) out of 50 of NGOs and United Nations agencies implementing CMAM programs in South Sudan (62%) participated in our study. Of the 31 agencies participating, 21 were CMAM implementing agencies (predominantly NGOs) and 10 were funding and policy level agencies (predominantly United Nations agencies). See Appendix 1.

### Scalability of the CMAM program

**Fig. 1:**
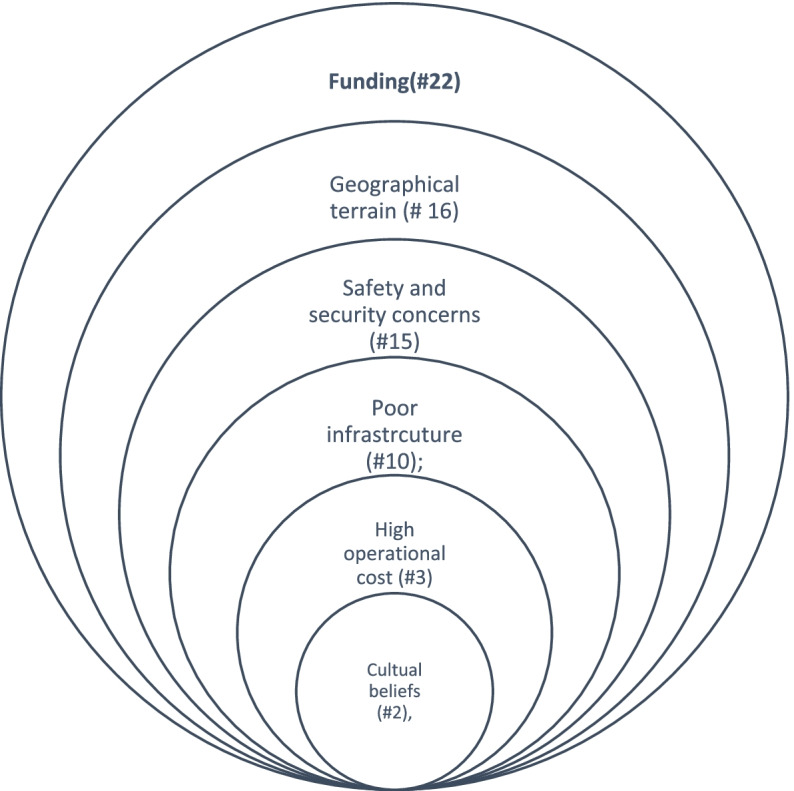
Key factors affecting the horizontal scalability of the program by number of cases referenced

**Fig. 2:**
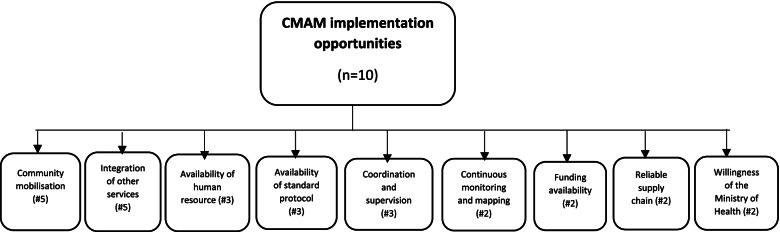
CMAM implementation opportunities by number of cases referenced

Scalability of CMAM was a top priority for almost all respondents from policy and implementation organisations. CMAM program scalability was mostly horizontal, with an emphasis on geographic distribution and coverage, operational expansion, program performance, and evaluation. Vertical scalability, which focuses on strengthening institutions and ownership through a sound policy, regulatory, and fiscal environment, received relatively little attention. Figures 1 and 2 are the summary of the key factors affecting the overall horizontal and vertical scalability of the program presented in order of intensity with the lowest in the innermost and by the number of respondents (cases) who mentioned the factor.

### Horizontal scalability

#### Constraints

Of the 31 respondents, 24 (77.4%) provided responses on constraints to effective horizontal scaling up of CMAM programs (Fig. 1). The main were funding (22/24); the geographical terrain of South Sudan (16/24); safety and security concerns throughout the country (15/24); the inadequacy of existing infrastructure for maximising scalability prospects (10/24); high operational costs (2/24); and cultural beliefs (1/24).

#### Funding

Two funding models emerged, each presenting a number of challenges. Participants noted that the first CMAM program funding model was largely through multi donor trust funds (MDTFs). Such funding mechanisms were unsustainable because of the current operational processes such as trends toward increased consolidation of funding streams and centralisation of funding mechanisms. For example, the funding was disbursed on “reimbursement modality” where implementing agencies request for funding after spending. The reimbursement modality also depended on implementing organisations’ ability to pre-finance their activities, which can potentially discourage organisational commitment to scalability. Participants also noted that the second funding model was through bilateral funding mechanisms between the government of South Sudan (25%) and multilateral donor organisations (75%). This funding model assumes that the government of South Sudan has a mechanism in place to meet its share of funding, which can be challenging given the state of political instability that has been shaking the country since its independence. The government of South Sudan’s inability to raise money locally to support the operational expansion of CMAM programs was further compounded by the administrative and reporting burden associated with dealing with different funding mechanisms. It was a general view among participants that, regardless of the funding model, externally funded initiatives focusing on nutrition specific, and nutrition sensitive interventions are most likely to be unsustainable. Local financial contribution is often overshadowed by external funding, a pattern that promotes dependence on external assistance. Participants also expressed a general fear of donor fatigue, especially for CMAM programs operating in a complex and a highly competing needs’ context such as is the case for South Sudan. As participants eloquently explained:

*The funding is Ad-hoc and unsustainable as it relies on external goodwill and willingness to support…..I don't expect this to be a sustainable way of funding either through UN agencies and other in country and other donors. South Sudan being in East Africa where conflict and other hazards are very common, competing priorities in the region are coming and donors may tend to reverse fund or hold for the new areas. Besides, South Sudan being in a very long humanitarian emergency, donors can get tired. Hence, the best way may be enabling the local government to own (star funding, staffing, monitoring) CMAM programs through frequent advocacy to senior officials and enabling local partners through working with international organizations”* [FR-13, policy]

*“The current funding modality is reimbursement system for cash and in kind for supply and other non-food items”* [FR-10, policy].

*In South Sudan, by far the largest funding mechanism is MDTFs, administered by the World Bank and the UN is responsible for a range of funding mechanisms [ with differing priorities and multiple stakeholders], including the South Sudan Humanitarian Fund (SSHF). Resource mobilization is informed through the Humanitarian Needs Overview (HNO) and the Humanitarian Response Plan (HRP) are generated annually by Clusters to serve both strategic and operational guidance for programming by [external] partners [which can be unsustainable]… Implementing partners (national and international NGOs) rely on funds or grants allocated by donors either directly or through targeted bilateral funds. However, the cost does not include many aspects, such as contribution from Civil Society Organizations (CSOs) partners or the procurement of RUTF using alternative funds, outside the central procurement and distribution through UNICEF* [FR-30, policy]

#### Geographic terrain

Participants described South Sudan as a vast geographical space with sparsely distributed population. Local communities are scattered across hard-to-reach mountainous areas, making travels difficult and a challenge. Due to the nature of the topography, most parts of South Sudan are often flooded which affects the road network and impedes access. The difficult geographical terrain discourages and threatens CMAM expansion to other areas of the country. For example, these participants recounted the challenges with the geographical terrain as:

*The vast areas with sparse population, this makes the community component of CMAM a problem, …poor road conditions as a result of flooding, this makes it hard to transport nutrition supplies and also hinders movement of beneficiaries* (FR-11, Implementation)*.*

*In accessibility due to natural disasters such as flooding during rainy seasons affected the implementation of the CMAM program* (FR – 12, Implementation)

*Poor road access and flooding during the rainy season, combined with the lack of secure storage for RUTF, meant that supplies must travel long distances by boat and by foot – increasing the time, cost and risk of programming* (FR – 22, Implementation)

#### Safety and security concerns

Closely related to the difficult terrain was the problem of insecurity across South Sudan. Participants identified three forms of insecurity that affect CMAM programs’ inception and uptake: revenge killing, political insecurity, and inter-communal conflict. The high level of insecurity harmed supply distribution, inhibited qualified workers from accepting offers and postings, and frequently led to community and beneficiary displacements. As participants summed it up:

*Insecurity caused by clan conflicts, revenge killings and worrying parts to the government. Most of the primary health care units are temporary structure, limited space making it extremely difficult to establish nutrition services* (FR-10, Policy)

*(politically motivated) as well as revenge killings and cattle raiding has displaced community members who have been traumatized year after year* (FR-17, implementation).

*the intra-communal violence has greatly affected the CMAM program implementation in South Sudan, nutrition supplies have been looted during intra-communal fighting and equipment destroyed. Most of the communities in need of CMAM services are not accessible due to targeted revenge killing, the local recruited staff can't move freely into the community”* [(FR-16, Implementation).

*Insecurity in some area where looting takes place, displacement of beneficiaries due to revenge killings and interclan fights…* (FR-11, Implementation)

#### Poor infrastructure

Participants consistently explained that a lack of adequate motorable roads, particularly during wet seasons, has harmed CMAM program implementation. Most health facilities in the communities needed CMAM services but were shutoff during the rainy season. Additionally, the lack of funds for the construction of storage facilities in remote nutrition locations has posed significant obstacles. The majority of CMAM sites have extremely limited storage areas that are incapable of storing six months’ worth of nutrition supplies. In certain places, there were insufficient healthcare facilities to support the operational expansion and implementation of CMAM programs Participants explained the problem of poor infrastructure as follows:

*The main challenge in South Sudan to implement CMAM was poor infrastructure that lead to high cost mainly of nutrition supply…* (FR-1, Policy)

*Poor infrastructure, lack of good road especially during rain seasons has affected CMAM implementation. Most of the facilities in the community in dare need of CMAM services are being cut off during season* (FR-16, Implementation)

*Poor local health infrastructure. The impact of the current conflict on health infrastructure in South Sudan surpasses that of the two-decade civil war that ended in Sudan’s independence. Where fighting has spread, health facilities have been destroyed* (FR-22, Implementation)

#### High operational cost

Participants highlighted the high operational cost including the cost of nutrition supply and transportation, For example, there were several circumstances that limited the adoption of ready-to-use therapeutic foods (RUTFs), which we grouped into three categories: supply-side, behavioural, and cultural. Our data revealed supply chain breakdown on multiple occasions, most notably during the wet season. It was costly to transport and store in remote places, where many beneficiaries lacked adequate storage. As a result, substantial logistical challenges arose that impacted delivery. In addition, some program beneficiaries sold RUTFs to purchase other household items or shared RUTFs with other children and family members. There have been reports of community members stealing the RUTFs from beneficiaries. There were expressed concerns about a lack of basic understanding regarding the use RUTFs. The mobility of some communities, particularly pastoralists who move from one location to another in quest of pasture caused some beneficiary households to discontinue the program. Some respondents recounted the high operational cost as:

*Increased cost of CMAM program in term of transportation of supplies, high volume of supplies needed….High cost of monitoring implementation,* (FR-23, Implementation)*.*

#### Cultural beliefs

Horizontal scalability was hampered by negative or harmful cultural practices in some communities. For example, participants explained that among some cultural groups, malnutrition was associated with the works of witchcrafts hence requiring spiritual and traditional solutions rather than using nutrition services. In other instances, participants noted that a few community members believe that the RUTFs cause diarrhoea, and as a result, they avoid using them. Culturally, most of the community members believed in traditional healing so they refused to use RUTFs to improve their children’s nutritional status. Hence, cultural beliefs and practices also affected the effective operational expansion of CMAM programs. Participants noted the role of cultural beliefs and traditional healing as:

*Some community members have the myth that the RUTF causes diarrhea…*(FR-12, Implementation), *most of the community members believed in traditional healing* [so they do not use RUTFs to treat malnutrition] (FR-16, Implementation)

*Poor cultural and poor behaviour that promotes negative deviance of service utilizations and adaptations*. (FR-27, Policy)

*Cultural practices in some communities affected the implementation of the CMAM program especially associating Malnutrition to witchcraft and looking for traditional or cultural ways of treatment…. Poor cultural and poor behaviours promote negative deviance of service utilizations and adaptations.* (FR-12, Implementation)

#### Opportunities

**Fig. 3:**
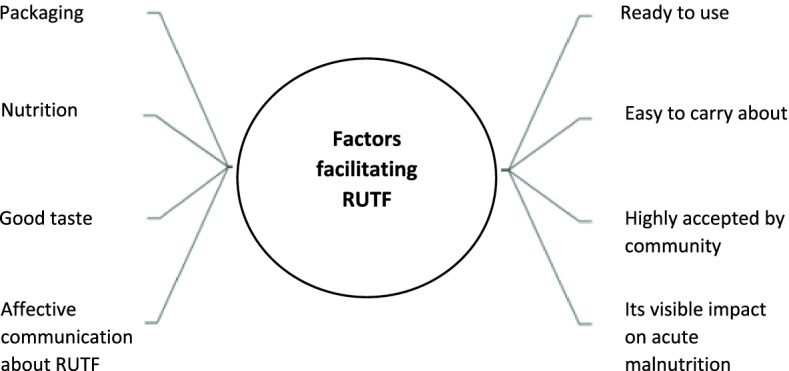
Factors facilitating RUTF in South Sudan

Strategies to maximize to maximize horizontal scalability of CMAM programs are summarized in Fig. 2. The first strategy centered on community mobilization to demystify cultural beliefs. Participants acknowledged that most community mobilisation activities in South Sudan have emphasized the therapeutic properties of RUTFs (Fig. 3). That is, in addition to having attractive packaging, the messages have predominantly focused on RUTFs having a good taste and being nutritious, a ready to use product, and easy to carry about with ease. Due to the effective community communication and advocacy about RUTFs, and through experience sharing and counselling by mother support groups and Community Nutrition Volunteers, community members at large have accepted the product. Other strategies to effectively support the horizontal scalability of CMA programs included ensuring adequate availability of competent human resources at the local level, integration of standard protocols into manuals at all level levels of service delivery which is supported by effective minoring and evaluation frameworks, financial sustainability built on local capacities (e.g. local capacities to raise program funds and procure RUTFs). Another important strategy noted by respondent on horizontal scalability was the opportunity to integrate targeted supplementary feeding programs (TSFP) and out-patient therapeutic program (OTP) into primary health care facilities. This integration resulted in increased accessibility to and continuation of services. A policy level respondent affirms this integration:

*Integrating OTP/TSFP programs in the same location has led to increased accessibility and continuity to nutrition services hence improved quality, efficiency, and effectiveness* (FR-24, Policy)

### Vertical scalability

#### Constraints

**Fig. 4:**
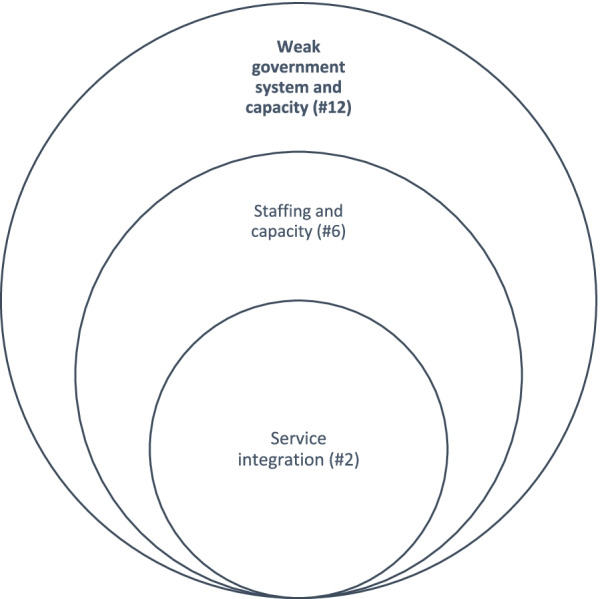
Key factors affecting the vertical scalability of the program by the number of cases referenced

Vertical scalability was somewhat weak in the data as compared to horizontal scalability. As can be seen from Fig. 4, the primary constraints to vertical scalability identified by the 19 of 31 participants (61%) who provided information were weak government system and capacity (12/19), staffing and capacity of program implementers (6/19), and service integration (2/19).

#### Weak government system and capacity

Politically, South Sudanese government has been at the forefront of developing policies and guidelines for CMAM implementation, monitoring, leading technical working group, providing mentoring, and supervision, coordinating, and supporting supervision (Table 1). However, there were concerns of inherent weaknesses in performing these roles effectively. The weaknesses in the government system made the operationalization of existing policies and guidelines very difficult. The main issues relate to the unstable political landscape characterized by inadequate staff and required skills and experience to lead key government roles and their poor coordination mechanism. Nutrition supplies are not part of the government’s essential drug list and there is limited or no budgetary allocation for nutrition programs by the government in national budgets and fiscal strategies. Participants explained this as:

…*gaps in coordination due to the fragility and stretched capacity of the South Sudan Government and Ministry of Health (MoH)* (FR-22, Implementation)

*Weak government health system for management CMAM activities as part of the health system - Government through MoH has no resource to lead the implementation of CMAM services* (FR-23, Implementation)

*Lack of existing government (MoH) policies and guidelines -Limited support in term of resources from MoH -Unstable government structures and systems (changes in leadership)* (FR-24, Policy)

**Table 1:** Key actors in the implementation and scalability of CMAM in South Sudan

Level of operation	Responsibilities
**Government**	
• South Sudan’s Federal Ministry of Health	• Develop policies and guidelines
• State Ministry of Health	• Lead technical working group and oversight
	• Monitoring and coordination
**UN Agencies**	
• World Food Programme	• Coordinate technical working group
• United Nations Children’s Fund	• Support the development of guidelines and policies
• Food and Agriculture Organization	• Compliance and monitoring
• United Nations Office for the Coordination of Humanitarian Affairs	• Direct implementation activities
	• Trainers/Master trainers
	• Funding advocacy
	• Joint field visit
	• Organise meetings for bilateral discussions
	• Develop corporate partner agreement
	• Supply chain management
	• Management of outpatient therapeutic program
	• Printing and provision office materials
	• Undertake gap analysis • Information management
	• Gap analysis
	• Rapid needs assessment
	• Reporting nutrition data
**NGOs and volunteers**	
• Action Against Hunger	• CMAM implementation and evaluation
• World Vision	• Case finding and screening
• International rescue Committee	• Community education
• Médecins Sans Fontières	• Referrals
• Welthungerhilfe	• Defaulter tracing
• Catholic Relief Services	• Community mobilisation
• Concern Worldwide	• Training of mothers and family members
• Plan International	• Home visit and follow-ups
• Universal Intervention and Development Organisation	• Counselling
• Grass Root Empowerment and Development Organization	• Village /community mapping
• Afro-Canadian Evangelical Mission	• Linkages with community and hospitals
• Save the Children	• Provide feedback on program activities
• ACROSS	• Program oversight at the community level
• Health Link South Sudan	
• Action For Development	
• Johanniter International Assistance	
• Plan International	
• Doctors with Africa	
• Amref Health Africa	
• Nile Hope	
• Andre Foods South Sudan	
• Alight	
• Mother and Children Development Aid	
• Relief International	
• AVSI Foundation	
• Joint Aid Management International	
• Help-Hilfe zur Selbsthilfe	

#### Staffing and capacity

As can be seen from Table 1, roles performed by all key actors – government, UN Agencies, and volunteers were overlapping. In some cases, except UN agencies that oversaw the funding and technical aspects of CMAM, it was unclear who was leading the process. UN agencies performed more of the identified roles (*n* = 19) compared to the government (*n* = 10) and volunteers (*n* = 12). UN agencies were mainly involved in technical coordination, support for the development of policies and guidelines, monitoring, funding advocacy, and bilateral discussions.

There was a shortage of skilled staff and capacity to facilitate the political scaling up of CMAM program. For example, the advocacy role of UN agencies has been directed more towards donor organisations for more funding without adequate attention directed towards government’s commitment to funding through a deliberate budgetary provisioning. Whereas there was evidence of advocacy activities from implementing agencies to UN agencies for additional funding, there was no report of advocacy directed at government. The key challenge for advocacy was the lack of funding for advocacy. This was evident in a statement by a participant alluding that there was *“no financial support advocacy and capacity building to support vertical scaling-up process including insufficient national advocacy*” (FR-3, Implementation). Another added “*Funding for capacity building remains the main [barrier] to vertical scaling up of the CMAM, in addition to the existing infrastructure and insecurity challenges* (FR-1, Policy).

#### Integration of services

Limited integration of services was the main challenge for organisational scaling up. Although the evidence shows community-based volunteers were involved in micro-level community implementation activities such as case finding and screening, community education, and referral, there was no clear evidence of their involvement at macro and policy level decision making processes. The lack of a clear government leadership in CMAM program implementation raises questions about ownership which also has implications for dependency on external assistance and scalability. Akin to the role of actors for scaling up CMAM programs was the political processes of coordination and supervision. The evidence shows that there is CMAM Technical working group which is co-led by the government, with UNICEF as the Nutrition cluster lead. The Technical Working Group is primarily responsible for standardising and modifying the country’s CMAM protocol and providing technical guidance and support to cluster partners and the government of South Sudan. The Nutrition Cluster remains the focal point for coordination of CMAM program through CSO partners. Most of the coordination occurred through biweekly and quarterly meetings. The coordination process begins at the community level, where community committees and implementing agency committees meet to discuss and make decisions about the need for CMAM programs within identified communities. At the county level, the Health Department facilitates health and nutrition coordination meetings, including discussions about the CMAM program. At national nutrition cluster level, decisions about the viability and impact of the CMAM program are made. South Sudan’s Ministry of Health also helps in this effort. Additionally, the existence of an inter-cluster coordination mechanism, a humanitarian coordination mechanism, nutrition technical working groups such as the CMAM program working group, and nutrition information working groups all contribute significantly to discussions and deliberations about technical/guideline details that contribute to achieving the CMAM program impact.

There was an acknowledgement from participants that investment in the development of a qualified human resource base to manage the CMAM programs has been a priority. The human resource development was supported by standards and protocols, and the Ministry of Health and implementing partners such as UNICEF provided opportunities for coordination and oversight. Whilst these efforts have contributed significantly to the current progress made in CMAM program implementation and impact, limited capacities at the government level, including high staff turnover remain a challenge for effective coordination. Some participants explained:

*High turnover in government offices with without proper hand over. Most of the technical personnel are no well facilitated by the government, affecting commitment and staff moral to deliver* (FR-10, Policy)

*Inadequate capacities for coordination especially at the national government -Inadequate resources (financial and human resources) -Lack of political will and commitment* - ( FR-29,Policy)

In terms of functional scaling up, the challenges centred on the limited funding diversification. As one participant noted:

*The funding especially for SAM with medical complication is limited and restricted that is not flexible enough to ensure coverage of all the interventions* (FR-28, Policy)

Another participant explained:

*The funding mechanism is mostly 'humanitarian' inclined and mainly an externally funded initiative which focuses on nutrition specific interventions. Such a mechanism is most likely unsustainable* (FR-29, Policy)

#### Opportunities

**Fig. 5:**
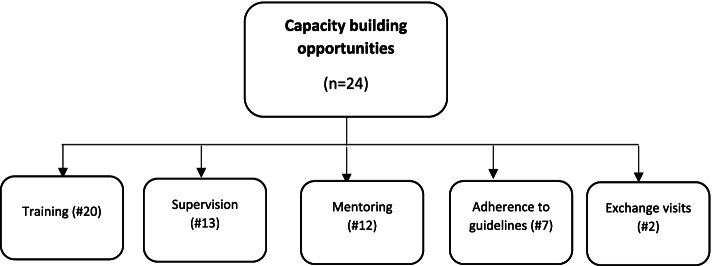
Capacity building as central to the implementation and scalability of CMAM by the number of cases

Our analysis identified five main opportunities for vertical scaling up of CMAM programs. They centred on capacity building opportunities for CMAM implementation partners and volunteers (Fig. 5). Training in CMAM program implementation (20/24) was the main capacity building opportunity. These training opportunities included several trainings of trainers (TOT), training workshops, and refresher training programs. A respondent expressed the nature and usefulness of these trainings as:

*At least one CMAM and MIYCN refresher training are organized in a certain geographic area every year that enable new staffs to update their knowledge and skill”* (respondent, implementation). Another expressed: *“Trainings of trainers were also organized for CMAM and MIYCN in the past three years that helps to get a master trainer for trainings organized at state level* (FR-5, implementation)

The second form of capacity building opportunities centred on supervision between various level of stakeholders and CMAM program implementation. Joint supervision-related training between the Ministry of Health and the project partners – mainly from the UN agencies are regularly carried out. However, our data show that supervision was targeted at the macro- (e.g. UN agencies, Ministry of Health, and NGO central headquarters) at the expense of the micro (e.g. supervisory activities and capacity of community members and CMAM program beneficiaries) and meso (e.g. field-level supervisory capacity of local and international NGOs the civil society or country/state) level. Other staff capacity building opportunities noted by respondents were mentoring, adherence to the CMAM guidelines and exchange visits. These training opportunities presented several advantages for CMAM implementation. They increased staff knowledge and skills, including record keeping and reporting abilities, and how this helped the smooth implementation of CMAM. However, due to the country’s low literacy level, participants noted that there is a need for ongoing capacity building, which can be a challenge given the urgent need to balance competing fiscal priorities.

For example, respondent said:

*The training enhanced the knowledge and skills of local staff to deliver quality service to the targeted vulnerable group in the community. The training enables the staff to keep good record of their works and make them produce good reports which can be used for many purposes. The staff were required to attend the CMAM training twice a year as per the national schedules. This makes the implementation runs smoothly and the expected results are being achieve* (FR-16, Implementation)

Another participant explained:

*The staff capacity building and training initiatives have improved the capacity of staff to manage CMAM programs hence improved performance of the programs and service delivery. However, there is need for continuous capacity building and trainings through trainings due to low literacy levels especially in the remote areas* (FR-19, Implementation)

Consolidating CMAM programs and integrating them into existing services result in streamlined activities, better coordination, increased quality of CMAM performance, reduced implementation cost, and everal positive impacts (Table 2). For example, integrating OTP/SFP programs in a single site has expanded access and continuity to nutrition services, resulting in improved quality, efficiency, and effectiveness. By involving community volunteers such as mothers’ support groups, community nutrition volunteer (CNVs), and home health promoters (HHPs) in encouraging breast feeding and other maternal, infant, and young child feeding nutrition (MIYCN) activities, the rate of acute malnutrition has decreased. Increased coverage (number of OTP/TSFP sites) in the community has also increased access to nutrition services, both preventive and curative, resulting in a reduction in malnutrition prevalence and mortality from malnutrition consequences. Household food security/nutrition status has improved because of community agents’ promotion of home-based small-scale diversified food production (kitchen gardening). Existing structures have been used to facilitate the implementation of CMAM programs to diversify their activities and maximise their synergy. For example, a respondent commented on the usefulness of the integration with other services in these words:

*Integration of CMAM into routine health care services e.g. malaria testing for all OTP admission has also led to early detection and treatment of malaria among children under which is also one of the leading cause of mortality in children. -Vit A and Micronutrients supplementation and deworming campaigns in CMAM programs has also led to reduction in prevalence of malnutrition and reduced burdens of common illnesses such as AWD.* [FR-24, Policy)

Home-based MUAC [mid-upper arm circumference] screening of children by trained caregivers and CNVs has increased not only early detection and referral of malnutrition cases, but also other prevalent child illnesses like as malaria, hence increasing overall health care utilisation. Additionally, integrating CMAM into normal health care services, such as malaria testing for all OTP admissions, has resulted in the early detection and treatment of malaria in children under the age of five, which is also a primary cause of child death. - Vitamin A and micronutrient supplementation, as well as deworming efforts, have also resulted in a decrease in the prevalence of malnutrition and a decrease in the burdens of common illnesses such as acute watery diarrhoea in CMAM programs. Despite the recognition of the integration of CMAM into existing health services, there were worries about the success of the integration process among both implementing and policy organisations, given the prevailing weakness in existing health care systems as well as weak government mechanisms incapable of providing program implementers with the essential assistance for the implementation and scalability. There were also concerns regarding the quality of staffing and human resources required to support scalability as well as the lack of a strong advocacy for the CMAM programs fiscal prioritisation. Participants were unanimous in their assessment of the CMAM programs’ insufficient policy and regulatory framework and the government machinery’s capacity to provide effective oversight.

**Table 2:** Project’s impact by the number of cases reported by policy level respondents for each theme

Projects impact	10 of 10 cases
Reduced mortality	8
Facilitated community ownership	6
Improved access to nutrition/reduced malnutrition	4
Early detection of nutritional support	3
Enhanced staff capacity	4
Increased healthcare service use	3
Enhanced economic outcomes for families	3
Improved coverage	2
Increased awareness of nutritional issues	2
Cost effective service	3
Improved knowledge of community members	3
Improved participation	1
Facilitate the integration of other programs	1

The involvement of local community-based structures and their capacity was central to vertical scalability of CMAM programs. All respondents who provided information on the community mobilisation confirmed the effectiveness of the CMAM community mobilisation approach, which was a catalyst for CMAM programs’ success. A respondent noted the significance of involving community structures in these words:

*The component of the community mobilization has been very effective in making the community to understand the CMAM program and improved the uptake of the services by the community. The community mobilization component has reduced the cases of defaulter and non-respondents as the years of implementation went by since the community are continuously being sensitized about the benefits of the program. The community also got involved in the monitoring the program especially the supplies which are meant for the treatment of their children. The community mobilization and sensitization has empowered the community with knowledge about malnutrition and how possible it is treating the children to recover. Volunteers were able to follow up cases and defaulters from the community and were able to refer them to the nutrition facilities. The advantage of selecting volunteers within the community is that, the community members could listen to the volunteers since they know them very well. This improved the home visiting by the volunteers to screen and follow up of cases”* (FR-12, Implementation).

### Emerging theory of change

**Fig. 6:**
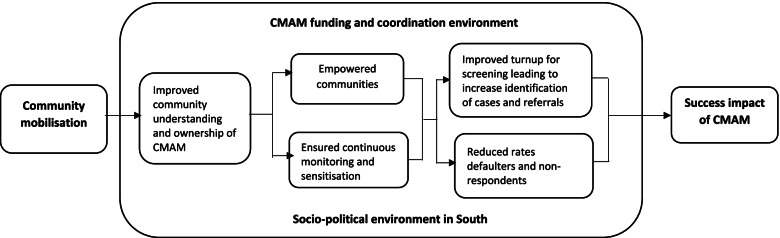
How community mobilisation contributes to the success of CMAM

Our analysis depicted the patterns and interactions between community mobilisation and CMAM program outcomes as critical components of the emerging theory of change in South Sudan (Fig. 6). The emerging theory suggests that the community mobilisation component has been extremely effective in increasing community awareness and a sense of ownership over community mobilisation procedures. The increased awareness and sense of ownership strengthened designated community structures and ensured continual monitoring and sensitisation of program beneficiaries. This resulted in an increase in screening turnout, which resulted in an increase in case identification and referrals. The rates of defaulters and non-respondents decreased, hence creating an opportunity for sustained treatment of malnourished children and subsequent impact.

## Discussion

The aim of this study was to identify pathway opportunities and approaches for horizontal and vertical scaling up of CMAM programs in South Sudan. We found that one major aspect of CMAM program scalability in South Sudan is the horizontal scaling up, focusing on protocol-driven replication of CMAM programs’ implementation and performance, operational expansion, and geographic distribution and coverage. A successful operational expansion of an intervention relies on the systematic use of evidence as well as well as the mobilisation of human, technical, and community resources [55]. This requires the assessment of the intervention effectiveness, the wide dissemination of emerging findings to facilitate the uptake such intervention (i.e. knowledge dissemination and advocacy), the provision of implementation standards and performance indicators (organizational processes), the description of intervention reach and potential adoption (i.e. costs and resource mobilization), and the intervention’s alignment with national and regional policy directions (i.e. monitoring and evaluation) [30, 55]. We found evidence of CMAM programs’ impact in South Sudan, including improving child recovery and saving thousands of lives each year. In the case of South Sudan, as is the case for other low and middle-income countries, CMAM program implementation, performance, and evaluation are guided by international standards and protocols such as the WHO protocols [12, 13] and the SHPERE Humanitarian Charter and Minimum Standards in Humanitarian Response [56]. In addition, CMAM programs have been extensively evaluated globally to establish their effectiveness [14, 20, 23–25], and have been found to achieve good recovery and survival rates, and low defaulting and relapse rates. The availability and wide dissemination of such evidence has led to unparalleled support for CMAM programs’ expansion by bilateral and multilateral donors. Similar findings have recently been reported in South Sudan [14], hence supporting their geographic and operational expansion.

However, we found that the horizontal scaling up of CMAM programs experienced several problems. We found that the efficiency, performance, and geographic coverage of the CMAM program operational expansion have been hampered by the rough geographical terrains, insecurity, poor infrastructure, and unsustainable funding models. South Sudan is a vast geographical space with sparsely distributed population, with local communities scattered across hard-to-reach mountainous areas, making travels highly risky. In 2020, the population density in South Sudan was 18 persons per square km (vs. 48 persons per square km in Suba-Saharan Africa) [57], making accessibility to health care facility and feeding programs difficult. In addition, we found that most parts of South Sudan are prone to flooding during wet seasons, have poor infrastructure (e.g. inadequate roads or extremely limited safe storage areas) and weak government systems and insufficient healthcare facilities to support CMAM program implementation, which, collectively affect the road network and impede access. A recent analysis of the spatial accessibility to basic public health services in South Sudan found that only 25.7% of the population live within one-hour walking time to a public health facility and only a quarter of the population (28.6%) lived within 5 km, whilst 16.8% public health facilities were non-functional due to the on-going civil conflict and insecurity [58]. These challenges continue to threaten the effectiveness of the operational expansion of CMAM programs, compounded by a high level of insecurity that triggers mass population displacements and hence affecting the speed of CMAM program inception and uptake as well as interrupting supply distribution and inhibiting qualified workers from accepting offers and postings. This could explain why, despite CMAM programs spreading to more than 80 countries and the number of children treated more than tripling since 2010, global acute malnutrition prevalence levels remain stubbornly high [14].

We found that the funding of CMAM programs in South Sudan has been ad hoc and unstainable, relying heavily of on external goodwill of international donors such as the MDTFs and bilateral funding. The MDTFs, whilst intended to enhance donor coherence, promote coordination and harmonisation, and reduce cost of CMAM program implementation, have many pitfalls. The pooled donor funding mechanisms create modalities driven by donors that centralize planning, coordination, and oversight. Available evidence points to disappointing results of the MDTF scheme including inter alia: poor and inflexible design and fund disbursements, inflexibility among donors and fund administrators, lack of understanding of political and socio-cultural contexts, a failure to cultivate and instil proper program ownership and institutionalisation, and donors’ reluctance to commit funds to local trust funds and associated failures resulting from prioritising harmonisation over strategic issues [59–61]. MDTFs are prone to the influence of media and other external factors (e.g. donors’ strategic geographic priorities), with substantial funds pledged when international and media interest is high, and then decreasing significantly as attention shifts [60]. There are also dual issues of capacity and state legitimacy, where national ownership and capacity are not evident. Although high-ranking recipient government officials may sit on the governance structure which oversees the MDTFs (i.e. steering committee), they are often circumvented by MDTF senior administrators [60]. As a result, in many cases, donor driven actions bypass state systems for the implementation of interventions, rather focusing and legitimising government-led identification of needs and priorities, with the net result being forgoing opportunities to build government capacity [59–61]. We found that South Sudan has to deal with MDTFs and bilateral funding, where the government of South Sudan accounts for 25% of the funding and multilateral donor organisations meet the remaining 75%. The government South Sudan is unable to mobilize the proposed 25%, further impacting CMAM program uptake and expansion. Dealing with two different funding models mean that the government of South Sudan reports to donors individually (e.g. bilateral and multilateral assistance) and collectively (MDTFs), creating an added layer of administration burden and bureaucracy. We also found that CMAM program funding was disbursed on reimbursement modality where implementers request for funding after spending, which threatens the viability of CMAM program scalability, especially given the complex disbursement process that implementers must navigate.

In terms of vertical scaling up, there was evidence of diversification of activities within CMAM programs beyond one function, with expanded numbers and types of activities to maximise synergy (functional scaling up). Cases in point include integrating OTP/SFP programs in a single site to expand access and continuity to nutrition services; integrating CMAM programs into primary health care facilities, CMAM programs’ incorporation of preventive measures such as counselling services, health education, and WASH programs; and the promotion of home-based small-scale diversified food production. These integrated services fostered positive behavioural changes that resulted in improved nutrition outcomes for families and communities to save children’s lives. There was also evidence of co-planning and co-coordination at the local, provincial, and state level (partial organisational scaling up). At the local level, community committees and implementing agency committees were established and met regularly to discuss and make decisions about the CMAM program implementation strategies. Despite these successes, beyond CMAM program delivery and associated implementation pathways, there was no evidence of lobbying and empowerment of the government to facilitate needed structural and institutional changes to address contextual, social, political, economic, and environmental challenges that impeded the ownership and institutionalisation of CMAM programs (political scaling up). Vertical scaling up was ad hoc rather than planned and budgeted for accordingly. We found that nutrition supplies are not part of the government’s essential drug list and there is limited or no budgetary allocation for nutrition programs by the government in national budgets and fiscal strategies. These challenges have been reported in other countries. In countries like Ethiopia, Malawi, and Niger that are known to have achieved widespread CMAM program scale-up to date through investing national budgets in CMAM programs at the state level, the amount of funds spent on CMAM programs by both donors and beneficiary countries is significantly low [62, 63]. Thirty-one Organisation for Economic Co-operation and Development and bilateral donors – including the European Union, the Bill & Melinda Gates Foundation and the Children’s Investment Fund Foundation currently spend less than 1% of their Official Development Assistance on ‘nutrition-specific’ interventions, which are the major nutrition component of CMAM programs. In addition, 24 high-burden countries allocate a meagre 1.7% of their general government expenditures to ‘nutrition-sensitive’ interventions (< 3% of government budgets recommended for domestic Governments to commit for nutrition), which are a key component for vertical scaling up of CMAM programs [63].

Whilst engaging local community, and provincial and state governments in coordination meetings and CMAM program planning improved relationships with different levels of governments and improved the program’s internal management capacity, there was no evidence of the government’s allocation of financial resources and financial self-sufficiency (diversification of resources) or enacted public legislation to earmark entitlements within the annual budgets (institutionalisation and ownership). For example, while the South Sudanese governments has been in the forefront of developing policies and guidelines for CMAM implementation, it cannot afford to fund their implementation and evaluation. Although respondents alluded to ownership of CMAM programs, local communities were limited to low level activities such as relying on community volunteers for community mobilisation, or case findings through home-based MUAC screening and referral of malnutrition cases. Most high-level activities such as Technical Working Group and the South Sudan Nutrition Cluster, securing needed funds from external donors, supervision, and capacity building of implementers, or securing CMAM program’s supplies were primarily targeting and undertaken by international NGOs and United Nations agencies. These findings are similar to those reported in Bangladesh. In a study assessing the preparedness of the health system to implement CMAM targeting children under-five years in sub-districts of Bangladesh, Kouam and colleagues [62] found that the government of Bangladesh developed CMAM guidelines and enacted policies offering free-of-charge health care for under-five children. However, the authors identifies five challenges that have hampered the effectiveness and scalability of CMAM programs in Bangladesh. Firstly, the implementation of CMAM programs was not under full government leadership. Secondly, three quarters of the funding (74%) were provided by donors for short-term interventions. Thirdly, half of the vacant positions (48.9%) in nutritional centres were unfilled. Fourthly, the health workers in nutritional centres at the local level were not trained in the management CMAM programs. Lastly, equipment and supplies failed to meet operational recommendations.

We found limited exit strategies in place, compounded by weak government systems and political instability, poor infrastructure, short-term humanitarian donor funding, high operational cost, and cultural beliefs (e.g. anything western is better than locally produced, high value of RUTF and likelihood of them being sold, and trust in traditional healing over RUTF to treat child malnutrition). Our findings suggest that vertical scaling up of CMAM programs requires government leadership, high political and public health priority, multidisciplinary and multi-sectorial coordination, increased policy commitment to tackling malnutrition, local resource mobilisation and financial sustainability, and embedding vertical scaling up into program planning and budgets for existing health systems, which is consistent with the literature [62, 63]. Local resource mobilisation and financial sustainability through policy, political, legal, budgetary and health systems changes are the key to institutionalisation, ownership, and mainstreaming of CMAM programs [63]. Such an approach requires United nations agencies and international NGOs to have clear exit strategies, as maximising greater domestic ownership of CMAM programs reduce significantly RUTF costs (as RUTFs are currently manufactured in and imported from advanced economies) and enable sustained investments in prevention strategies [24, 63–67].

## Limitations

Qualitative surveys delivered via online survey software are self-administered and predominantly use a series of questions that participants can answer in their own words through textual responses Data collection is relatively quick, cheap, and there is no need for transcription [45]. However, online qualitative surveys are prone to sampling bias, including reporting bias. For example, respondents may search the internet or consult other materials when answering question rather than using their own knowledge and experience. Another limitation of online qualitative surveys is the lack of non-verbal information encountered in a face-to-face context (e.g. tone of voice and body language), which enrich the truthfulness of data collected. While textual paralanguage, that is, written expression of nonverbal audible, tactile, and visual elements that supplement written texts such as words they choose to emphasize their points, symbols, images, punctuation, demarcations can be used to convey personal reaction [68], these were at the discretion of the respondents and the Qualtrics software has limitations on these characters. Nonetheless, textual response are more explicit and visible than often subtle non-verbal face-to-face cues, which are only subconsciously picked up [68–70].

## Conclusion

Notwithstanding the above limitations, our findings suggest that CMAM programs are of utmost significance for children’s survival in South Sudan. Whilst CMAM programs are supported by well developed sets of principles, protocols, and minimum humanitarian standards, our findings suggest that considerations need to be made for context-specific factors to better plan for the effective delivery mechanisms and incremental scalability pathways of these interventions. Similarly, the future of CMAM programs remain uncertain given the lack of ownership and limited financial and human capacities. It is a policy imperative to advocate for strengthening domestic capacity and resource mobilization while leveraging external collaborations. The ad hoc and unstainable funding model of CMAM programs in South Sudan means that their costing mechanisms are underdeveloped. CMAM programs in South Sudan may require an adequate costed scaling up model that takes into account both donor and domestic funds. The domestic funds could be provided through a sliding scale of match funding percentages with domestic resources increasing with time, supported by a monitoring framework for financial tracking of domestic CMAM budgets and their significance. Therefore, there is an urgent need for stronger and continued engagement with the government of South Sudan through lobbying and targeted communication strategies to make budgetary commitments towards overhauling the health infrastructure and improve its operational efficiencies to better support both horizontal and vertical scaling up of nutrition interventions. Such fiscal strategies need to be supported by a long-term plan with clear exit strategies to build and strengthen nutrition systems, with CMAM programs becoming a critical service under the universal coverage of the South Sundanese health system. The nutrition system strengthening should also encompass scoping and nutrition capacity assessment of pre-service curriculum for academic institutions including tertiary colleges and universities providing nutrition courses in line with global trends and innovations in nutrition interventions. In addition, the government of South Sudan needs to consider including nutrition supplies especially RUTF, ready-to-use supplementary foods, and other therapeutic products into the ministry of health’s list of essential drug for strategic positioning, visibility, and prioritization of nutrition services. Finally, the South Sudanese government needs to improve public safety generally, provide a guaranteed security of health facilities, invest in infrastructure to reduce CMAM programs’ operational costs, and implement a tested social and behaviour change communication framework to address cultural beliefs that hinder vertical integration.

## Supplementary Information

**Additional file 1: Appendix 1.**

## Data Availability

Provided.

## Abbreviations

CMAM: Community-based Management of Acute malnutrition
CNVs: Community nutrition volunteer
HHP: Home health promoters
LMICs: Low and middle income countries
MIYCN: Maternal, infant, and young child feeding nutrition
MDTFs: Multi donor trust funds
MSF: Médecins Sans Frontières
MUAC: Middle upper arm circumference
NGOs: Non-government Organisations
OTP: Outpatient Therapeutic Program
RE-AIM: Reach, effectiveness, adoption, implementation, maintenance
RUTF: Ready-to-use Therapeutic food
WHO: World Health Organisation
