# Supplementary material for: Pathways and approaches for scaling-up of community-based management of acute malnutrition programs through the lens of complex adaptive systems in South Sudan
Source: Arch Public Health. 2022 Sep 5;80:203. doi: 10.1186/s13690-022-00934-y (PMC9442594; doi:10.1186/s13690-022-00934-y)
Supplement: Supplementary file 1 — Additional file 1: Appendix 1. [file 13690_2022_934_MOESM1_ESM.docx]

Appendix 1. Participants and their level of occupation by gender

| **Participant ID** | **Occupational level** | **Gender** |
| --- | --- | --- |
|  |  |  |
| FR1 | Policy | Male |
| FR2 | Implementation | Female |
| FR3 | Implementation | Male |
| FR4 | Policy | Female |
| FR5 | Implementation | Male |
| FR6 | Implementation | Female |
| FR7 | Implementation | Female |
| FR8 | Implementation | Female |
| FR9 | Implementation | Female |
| FR10 | Policy | Male |
| FR11 | Implementation | Female |
| FR12 | Implementation | Male |
| FR13 | Policy | Male |
| FR14 | Implementation | Male |
| FR15 | Policy | Male |
| FR16 | Implementation | Male |
| FR17 | Implementation | Male |
| FR18 | Implementation | Female |
| FR19 | Implementation | Female |
| FR20 | Implementation | Female |
| FR21 | Implementation | Male |
| FR22 | Implementation | Female |
| FR23 | Implementation | Male |
| FR24 | Policy | Male |
| FR25 | Implementation | Female |
| FR26 | Implementation | Female |
| FR27 | Policy | Female |
| FR28 | Policy | Male |
| FR29 | Policy | Female |
| FR30 | Policy | Male |
| FR31 | Implementation | Male |
